# STOPFLU: is it possible to reduce the number of days off in office work by improved hand-hygiene?

**DOI:** 10.1186/1745-6215-11-69

**Published:** 2010-06-04

**Authors:** Carita Savolainen-Kopra, Jaason Haapakoski, Piia A Peltola, Thedi Ziegler, Terttu Korpela, Pirjo Anttila, Ali Amiryousefi, Pentti Huovinen, Markku Huvinen, Heikki Noronen, Pia Riikkala, Merja Roivainen, Petri Ruutu, Juha Teirilä, Erkki Vartiainen, Tapani Hovi

**Affiliations:** 1 National Institute for Health and Welfare (THL), Department of Infectious Disease Surveillance and Control, Gastrointestinal Infections Unit, Helsinki, Finland; 2 National Institute for Health and Welfare (THL), Information Department, Information Systems Development and Support Unit, Helsinki, Finland; 3 National Institute for Health and Welfare (THL), Department of Vaccination and Immune Protection, Vaccine Research Unit, Helsinki, Finland; 4 National Institute for Health and Welfare (THL), Department of Vaccination and Immune Protection, Viral Infections Unit, Helsinki, Finland; 5 Kesko Oyj, Helsinki, Finland; 6 Department of Mathematics and Statistics, University of Helsinki, Helsinki, Finland; 7 National Institute for Health and Welfare (THL), Division of Health Protection, Helsinki, Finland; 8 Outokumpu Oyj and Outotec Oyj, Espoo, Finland; 9 Nordea Bank Finland Plc, Helsinki, Finland; 10 National Institute for Health and Welfare (THL), Department of Infectious Disease Surveillance and Control, Helsinki, Finland; 11 SOK, Helsinki, Finland; 12 National Institute for Health and Welfare (THL), Division of Welfare and Health Promotion, Helsinki, Finland

## Abstract

**Background:**

Acute infectious diseases are major causes of short periods of days off from work, day care and school. These diseases are mainly caused by viruses and hands have a key role in their transmission. Thus, hypothetically, they can be controlled with means of intensified hand hygiene. In this study we aim to elucidate the effect of acute infectious diseases on the work contribution in common office work and study the influence of improved hand hygiene on possible reduction of infectious disease episodes and days off from work due to acute infectious diseases.

**Design:**

The voluntary participants have been recruited from six companies in the Helsinki region. The designated 21 study clusters were identified as operationally distinct working units each containing at least 50 people. The clusters were matched and randomized based on results of a pre-trial contagion risk survey. Improved hand hygiene is being executed with guided hand-washing with soap and water in one intervention arm and with alcohol based hand rubbing disinfectant in the other. Participants in both arms have received guidance on how to avoid infections and how to implement contagion stopping habits. A control arm is acting as before regarding hand hygiene. Data collection for evaluation of the efficacy of the interventions is based on self-reporting through weekly electronic reports. The questionnaire is enquiring about possible respiratory or gastrointestinal symptoms during the preceding week, and requests a daily report of presence of symptoms and working capacity. Etiology of the symptoms is not searched for individually, but contribution of different viruses is evaluated by sentinel surveillance, where occupational health clinics located in the premises of the participating companies collect specimens from employees visiting the clinic. Common causative agents of the diseases are being searched for using real-time PCR techniques. The duration of the intervention will be 16 months. Primary endpoints of the study are the number of reported infection episodes in a cluster within a time frame of 100 reporting weeks and the number of reported sick leave episodes in a cluster within a time frame of 100 reporting weeks.

**Trial Registration:**

ClinicalTrials.gov Identifier: NCT00821509

## Background

**Figure 1:**
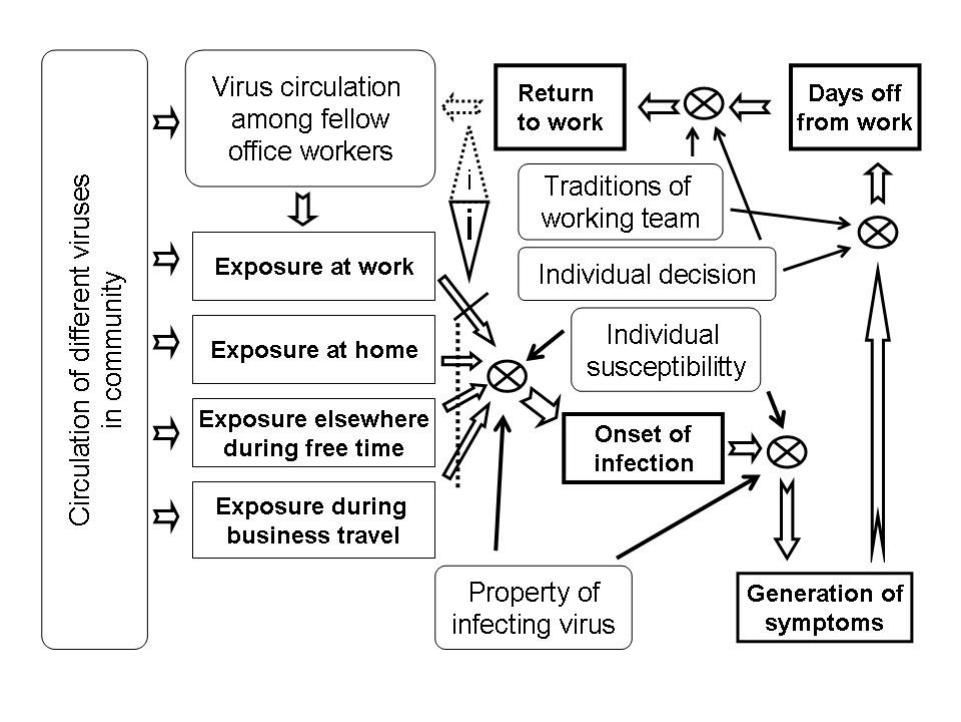
**Factors influencing viral infection induced periods of days off from work**. Steps in the process, from the point of view of an individual, are indicated in boldface by squares connected anticlockwise with each other by open arrows. Factors influencing translocation from one step to the following are shown in rounded squares and by black arrows to "regulation points" (crossed circle). Dashed open arrow represents contribution of viral load to the working team in case an infected individual remains at work or returns from home before the excretion period is over. Back-to-back triangles with **i **inside indicate the site of influence of improved hand hygiene at work and that of transmission-limiting behavior at work.

Respiratory tract infections (RTI) cause a large part of short periods of days off from work, day care and school. Gastrointestinal tract infections (GTI) are rarer in the working population, with sporadic norovirus or rotavirus outbreaks excluded. GTIs are, however, common in children, thus possibly leading to guardians' absence from work. Parents of children attending day care centers or in school are prone to RTIs and GTIs more often than other working population [1]. Often, because of the generally mild nature of the diseases, infected people come to work in spite of symptoms, and may therefore initiate transmission of infection among other workers. The chain of events from exposure to a pathogenic microbe to subsequent infection and generation of symptoms affecting working capacity is a very complicated one, and is influenced by many factors potentially causing variation, such as the multitude of causative agents, different natural immunities to the agents, and different individual histories of infections (Figure 1).

Acute RTIs are mainly caused by viruses. They are spread via excretions of the carrier with or without symptoms to respiratory tract, mouth or conjunctiva of a susceptible person. Viral GTIs are also spread via contaminated food or water. At least RTIs may be further spread via droplets or aerosols generated by coughing or sneezing. Hands have an essential role in the transmission; from direct contacts to contagion via contact surfaces, such as door knobs, taps in toilets, shared utility articles, table surfaces, to respiratory tract, mouth and eyes [2].

The effect of hand hygiene and advised behavior aimed at limiting the transmission of infections has been described in several research reports [3,4]. In recent meta-analyses, however, large part of the publications have been considered deficient in a way or an other, and due to differences in settings and variables affecting the final results comparison of the accepted studies has been problematic [5,6]. Based on earlier research and meta-analyses it seems, that improved hand hygiene may help in limiting the spread of infections in "semi-closed" environments with high infection pressure, especially in hospitals, but also in day care centers [7,8], schools [9] and military service [10]. In regular office work this has not been studied. Even though the annual epidemics of both RTI and GTI are familiar to everyone, there is little research based knowledge on the effect of acute infectious diseases on the work contribution in a defined working community, except in health care, children's day care and schools. One of the goals of this study is to elucidate the effect of acute infectious diseases on the work contribution in common office work.

In order to stop the spreading of difficult bacterial infections, alcohol based hand disinfectants are often used in hospitals instead of more traditional hand washing. In addition to the efficacy and the ease of use, the conversion to rub use has been justified e.g. with reduced total cost in a hospital setting [11]. Short treatment with alcohol does not, however, destroy all viruses, and there are reports suggesting that the spreading of the most common viral infections could be stopped with careful hand washing with soap and water at least equally well [6]. Hence, this study was designed to contain two intervention arms exploiting intensified hand-hygiene, one using water and soap, the other mainly an alcohol-based disinfectant. In this paper we describe the general design of the trial, try to justify the compromises we decided to make in the design in order to get the study going, and discuss obvious and potential weaknesses of the study some of which emerged only after launching the interventions.

## Methods

### Aim of the study and general description

This study is aiming at investigating the effect of improved hand hygiene on possible reduction of days off from work due to acute infectious diseases. The study is not designed to compare the two different hand cleaning techniques *per se *but includes two independent assessments of the combined efficacy of the advised transmission limiting behavior and intensified hand cleaning on the occurrence of RTI and GTI and consequent periods of days off from work in a common office work. Data collection for evaluation of the efficacy of the interventions is based on self-reporting through Monday-morning electronic reports using a simple standard format. The questionnaire is enquiring about possible symptoms of RTI or GTI during the preceding week, and requests a daily report (including weekends and other holidays) of presence of symptoms and working capacity. Etiology of the symptoms will not be searched for on individual basis in this study but a connection to concurrent RTI virus epidemiology is built by a sentinel surveillance, where occupational health clinics located in the premises of the participating companies send a standard number of weekly specimens collected from employees visiting the clinic. For possible GTI outbreaks a standard outbreak investigation principle will be followed, including 3-5 specimens from typical patients. Common causative agents of the diseases will be searched for using real-time PCR techniques. The duration of the intervention will be 16 months.

### Setting, identification of participating clusters and recruitment

Recruitment of participants was targeted to six companies in the Helsinki Region each including an occupational health clinic of their own. The participating companies include two leading retailers in Finland, two banks, a mining and metallurgical technology provider and a stainless steel producer. Only units involved in common office work were included. The premises of all companies were of the same kind including both open-plan offices and personal office rooms to some employees.

Participants were recruited in 21 operationally distinct volunteer groups, later referred to as study clusters, each including at least 50 persons. Operationally distinct means that members in a cluster had a likelihood of meeting other members of the same cluster during the daily working hours and shared certain physical spaces such as working room, toilets and/or refreshment/resting corners. Members of another cluster did not share these things but, in some cases, might be occasionally met, e.g., in a company restaurant.

Information about the purpose of the study and expected consequences of participating in it were given through different channels including posters and hand-outs, informative talks at staff meetings, and on company intranet web pages. Finally, everybody working in an identified cluster was sent an email requesting to answer a questionnaire about transmission risks (see below) and offering the possibility to participate in the study by weekly email reporting. Inclusion criteria were "Volunteers working in defined units". Exclusion criteria were "Persons with open wounds or chronic eczema in hands".

### Cluster matching and randomization

To minimize the effect of confounding factors the intervention clusters were matched and randomized prior to onset of the interventions. A questionnaire enquiring, among other things, about numbers and ages of children, their possible out-of-home day care, personal properties such as smoking, chronic diseases, and potential differences in contacts to other people during the daily work, was sent by email to all employees of the selected clusters (Additional file 1). An arbitrary virus transmission risk score was calculated for each cluster based on the results of these potential participant questionnaires [12]. First, the occurrence of each risk factor in a given cluster in relation to the frequency of the factor in the entire population interviewed was assessed. Next, these ratios were inter-connected to reveal an arbitrary transmission risk sum score for the cluster using the following formula:

Indicator variables:

x1 school aged or younger children

x2 at least one child under school age attending day care/pre-school classes/weekly club

x3 smoking (0 = if 1 or 2, otherwise > 1, see questionnaire in Appendix 1)

x4 medically diagnosed chronic heart condition, atherosclerosis, asthma or other respiratory disease

x5 mainly use of public transportation for commuting to work

x6 grown-up exposed to children with cold or vomiting/diarrhea disease at work in school, kindergarten, children's club, health care etc.

where s1-s6 denote corresponding unit-wise sums of each indicator, m1-m6 corresponding total sums over all clusters, i.e. border sums describing frequency of a given variable in the complete material and n, number of people in the cluster.

As can be judged from the above, living with pre-school children in day-care was considered the relatively strongest risk factor and the corresponding risk ratio multiplied by two in the formula. The clusters were then divided in groups of three most similar based on the calculated scores, and members of each triplet were randomized in one of the two intervention arms or in the control arm.

### Intervention arms and arm-specific guidance of hand hygiene and behavioral habits

A total of seven office units (247 reporting persons) belonged to intervention arm 1 (IR1), in which the intervention was based on hand-washing with soap and water. Further seven office units (199 reporting persons) belonged to intervention arm 2 (IR2), the alcohol disinfectant group. The remaining seven units (218 reporting persons) served as a control arm (IR0). The interventions were planned to continue more than 12 months, preferably one and a half year, in order to cover various season-dependent viral epidemics.

Improved hand hygiene was executed with guided hand-washing with soap and water in one intervention arm and with alcohol based hand rubbing disinfectant in the other. Participants in both arms also received identical guidance on and demonstration of how to avoid infections and how to implement contagion stopping habits. The guidance included instructions for proper coughing and sneezing, for avoiding hand shaking when possible, and frequent hand washing in office and at home. The guidance was given personally to all participants by a specifically trained research nurse and each participant also received written instructions for further reference. The participants in the intervention arms received free of charge containers of the relevant soap or disinfectant, respectively, to be used at home. Participants in the intervention arm 2 also received free of charge containers of disinfectant for personal use in the office. Participants in the control arm were advised to act as before regarding hand hygiene. Toilets and other hand washing spots in all participating clusters were equipped with the same soap, Erisan Nonsid Farmos, Finland, for hand washing. The alcohol containing hand rubbing disinfectant used was LV Käsihuuhde Berner, Finland.

The study was started in two steps following the progression of recruitment. The first batch included 12 units including 374 volunteers in total. The units were matched and randomized together to form four triplets. The second step included 9 units with 306 volunteers in total. These were matched and randomized together to form three triplets. The mean participation percentage in work units was 32.4%, which is reasonable given the large intra-class correlation [13] in the cluster expected in the study.

### Weekly reporting of exposure to infectious agent carriers and of day-by-day symptoms of infectious diseases and working capacity

The participants in all study arms are reporting weekly exposure to infectious diseases and own possible RTI or GTI symptoms and related days off work through internet. Every Monday the participants receive via email a link to an electronic questionnaire (Additional file 2). Design of the questionnaire, sending of the weekly calls for reporting, and replies containing database were executed with a commercially available provider of software services for feedback management and data collection Digium, Digium Enterprises, Espoo, Finland). The collected data is then further stored in an in-house database for monitoring and analyses.

### Drop-outs and post-onset enrollment of new participants

It was expected that some participants will give up weekly reporting of symptoms and days-off for various reasons or are, for operational purposes of the employer, translocated from their study cluster or leave the participating company. On the other hand, it was expected that before the end of the trial also new people are recruited by the employer to the study clusters. It was decided that since the follow-up week-based data will be primarily analyzed in a cluster specific way rather than targeting to individual participants, part time reports could be included in the data to be collected without problems. Likewise, individuals changing the study arms during the trial, first will provide data to the first arm, and after personal instructions relevant to the new arm, start to report to the other arm. Similarly, new recruits in the study clusters will be offered the possibility to participate in the trial and, after receiving training and instructions, will start reporting accordingly.

### Facilitation of interventions and motivation of participants to adherence in trial

The implementations of the interventions are being monitored during monthly check up visits by a research nurse to the participating office units. During the visits the research nurse checks the availability of hand soap and/or alcohol disinfectant products, receives comments on weekly reporting and supports participants in continuation and commitment to the hand hygiene behavior of the unit in order to increase compliance and minimize loss-to-follow-up. Any new employees volunteering to participate in the study will also receive guidance to intensified hand hygiene and behavioral habits during the visits. The participants are receiving monthly an electronical popularized information spot concerning viral diseases as an incentive and motivation to maintain agreed hand hygiene and behavioral habits.

### Evaluation of adherence to instructions

There is no way to directly measure whether the participants of this kind of an intervention study in real life follow the given instructions. Some indirect ways were considered on top of the sporadic observational information obtained during the monthly visits of the research nurse to the cluster sites. First, we wanted to monitor the usage of soap and disinfectant cluster-wise at the work place and individually at home. However, it turned out that this was not feasible, because of complex organization of the maintenance of the toilet and washing facilities in the participating companies. So, we were left with another questionnaire, a self-reporting survey of hand hygiene behavior. A survey to study hand hygiene behavior was executed among the volunteers in the beginning of the study, in January-February 2009, before the results of the contagion risk survey were published and the intervention arms designated (Additional file 3). The survey was planned to be repeated at least in the end of the study. The results of the surveys will also be used to evaluate comparability of clusters within the triplets and of the intervention arms.

### Monitoring of concurrent virus circulation in the source population

It was decided that no attempt will be made to find out the etiology of every reported infection episode. Samples for virological analysis are being collected to monitor circulation of agents causing respiratory and gastrointestinal complaints in the participating companies. Participants of the study were not especially encouraged to deliver samples for virological analysis. Rather, samples are being retrieved from any patients with respiratory or gastrointestinal symptoms attending the occupational health clinics located in the premises of the participating companies, thus originating also from people working in company units not included in the study. The seven participating occupational health clinics were advised to collect 2-3 respiratory samples per week. Fecal samples from gastrointestinal patients were advised to be taken when an outbreak was suspected, up to five specimens per outbreak.

Nasal and pharyngeal stick samples from patients with respiratory symptoms are taken by standard techniques. They will be analyzed with validated real-time polymerase chain reaction (PCR) techniques for the following viruses: influenza viruses A and B, respiratory syncytial virus, parainfluenza viruses 1, 2, and 3, adenoviruses, human rhinoviruses and human enteroviruses. Fecal samples will be taken from patients with gastrointestinal symptoms. They will be tested for noroviruses with validated real-time reverse transcriptase (RT)-PCR technique.

### Ethical issues

Participation in the study is voluntary to office employees. At the time of recruitment all potential participants received an electronical information sheet of the study, at the end of which the willingness to participate was asked. Informed consent for virological sample analysis is being obtained from patients visiting occupational health clinics because of respiratory or gastrointestinal symptoms.

The person register formed in the study is being stored in locked facilities and in secured electronic files. Personal data collected was limited to email address and working unit. Privacy protection is ensured with use of secured internet connections for data collection. The results of virological sample analysis will not be connected to results of self reporting at personal level.

The study has been approved by the Institutional Review Board of the National Public Health Institute, Helsinki, Finland (tusu460 9/2008). The study has been registered in Clinical Trials.gov Protocol Registration System, ID: NCT00821509.

### Study timeline

The study was started in June 2008 with the design and recruitment of participating companies. The contagion risk surveys were executed in January-February 2009. The interventions and data collection were started in February-March 2009. The interventions are planned to run 16 months (64 weeks) to cover the seasonal variation in the epidemics of the causative agents. The interventions will end in May 2010. The data analysis for the primary outcomes will start in June 2010 and is planned to end in December 2010. The total duration of the study will be two years and seven months. Additional analyses described will continue after that.

### Project funding

The study is being funded by the National Institute for Health and Welfare, Helsinki, Finland (former National Public Health Institute, Helsinki, Finland) and the Finnish Work Environment Fund, grant number 108306.

### Project administration

The project group takes care of data collection and monitoring, intervention monitoring and coordination of the sample collection and analysis. The steering committee of the study meets approximately every two months to oversee the implementation of study plan, to coordinate the interventions and to consider the conclusions of the project group.

## Results

### Data processing and basic definitions

The raw data on occurrence of RTI and GTI symptoms and associated days-off from work will be collected individually day by day but reported in blocks of seven days i.e. on weekly basis. For each cluster, the individual weekly reports will be summed together to get a number of total follow up weeks in a given cluster. Another summing up will be made for each calendar month in order to be able to analyze seasonal variation of infections and periods of sick leave from work. Individual weekly reports will be fused together to form a continuum of dates for each individual. These datasets will then be used to determine the numbers and lengths of episodes with RTI or GTI symptoms and those of associated sick leaves.

An ***infection episode ***was defined as a continuous period of time with respiratory and/or GT symptoms reported on successive days. However, in the case of a one-day interval without symptoms between symptomatic days, the flanking days with symptoms on both sides of the interval will be considered one single infection episode and the day without reported symptoms will be counted into the designated length of the episode. In the case of an interval of two or more symptom-free days, the flanking infection episodes will be considered distinct. A ***RTI episode ***and a ***GTI episode ***was determined in a similar way based on days with reported RT or GT symptoms, respectively. A ***sick leave episode ***associated with a RTI, GTI or an infection episode was defined as a continuum of successive days absent from work because of own RTI, GTI or either symptoms, respectively. The days with partial absence from work will be included in the sick leave periods. Days of absence from work flanking a week-end or other holiday of maximally two days will be included in a single sick leave episode provided that the symptoms continue through the intervening days. An ***absence episode ***due to infectious disease was defined as a continuous period of time absent from work due to own or child's RTI or GTI. Table 1 shows the links of these definitions to the alternatives in the weekly report.

**Table 1:** Linkage of study definitions to the weekly report.

	**"Symptoms" mean symptoms of respiratory infection or vomiting/diarrhea disease**							
	**Healthy at work or out of work as designed**	**At work with symptoms**	**Symptoms, but not supposed to be at work**	**Part of the day at work with symptoms**	**Absent due to symptoms**	**Absent due to child's symptoms; own symptoms as well**	**Absent due to child's symptoms; self healthy**	**Other reason for absence**
	
Infection episode*		x	x	x	x	x		
Sick leave episode				x	x			
Absence episode				x	x	x	x	

* RTI and/or GTI episodes will be defined the same way based on reported RTI and/or GTI symptoms

### Evaluation of matching and comparability between the arms

As the participants in the study clusters evidently will partially change during the course of the trial, because of drop-outs and new recruitments, the matching of the clusters within each triplet and the comparability between the intervention arms will be confirmed

a) by repeating at the end of the trial the questionnaire-based interview inquiring individual transmission risks (the questionnaire that was used for matching).

b) by assessing the number of participating persons in each cluster at six month intervals: the variation (the number of drop-outs and new recruits) will be compared between the clusters in each triplet and between the intervention arms.

c) by assessing similarity of results in the evaluation of the intervention implementation between the clusters in each triplet and between the intervention arms (survey for hand hygiene behavior).

### Study endpoints and outcome measures

#### Primary endpoints

a) the number of reported infection episodes in a cluster within a time frame of 100 reporting weeks

b) the number of reported sick leave episodes in a cluster within a time frame of 100 reporting weeks.

#### Outcome measures

The results will be analyzed comparing the control arm to intervention arm 1 and intervention arm 2, respectively.

The means and standard errors of the numbers of illness and absence episodes in each cluster will be calculated. The efficacy of the two intervention modes will be evaluated separately comparing the corresponding values of invention arm 1 and intervention arm 2 to those of the control arm. The setting will also enable comparisons between intervention arms 1 and 2. Classical hypothesis testing, both for parametric and non-parametric setting and adapted for cluster-randomised trials, with plausible assumption about the data will be launched e.g. similar to that described by [14]. Yet, for getting a probabilistic view towards the study parameters for each arm and every cluster in them, we will exploit the Bayesian approach [15]. With defining our current knowledge of the parameters as a priori and merging it with the likelihood of the data, we will obtain the posterior that will enable us to have a broader view on the study parameters, to see the differences of parameters in each arms and clusters, not only with some sharp frequency tests but also with the probability scope, in order to make inferences e.g. about the likelihood of each parameter to exist in any given interval for the whole population. Due to several person-dependent variables influencing especially the number of sick leaves, this comparison will provide the essential result of the study.

Effects of the interventions on the occurrence of respiratory infections and vomiting/diarrhea diseases will be analyzed both separately and together.

This study will evaluate the efficacy of the entire intervention procedure, not only that of hand washing method, albeit that is where the difference between the two intervention groups lie. The presumption is that based on analyzed self-reports concerning own illness control arm, IR0 ≠ intervention arm, IR1~ intervention arm, IR2. In previous studies performed in semi-closed populations the difference has been 10-50%. Here the default value is closer to the lower than the upper margin of the range.

### Secondary end points and outcome measures

a) the number of days with reported symptoms of RTI and/or GTI in a cluster within a time frame of 100 reporting weeks

b) the number of days-off due to own RTI or GTI in a cluster within a time frame of 100 reporting weeks

Evaluation of significance and presumptions will be the same as in primary outcome measures. As the implemented intervention presumably affects the probability to fall ill on the first hand and not so much on the duration of the illness, the potential differences in this comparison may be smaller than when using the primary end points

### Other analyses

#### Overall impact of infectious diseases on work contribution and the modulating efficacy of the interventions

In addition to the aspects in the primary and secondary outcome measures this will include absences due to RTI or GTI of dependant. Analyses similar to those described above for *periods of sick leaves *will be carried out using the *periods of absence *as starting point.

#### Transmission routes

Suspected origin of contagion will be analyzed by connecting the exposure recognized by the reporting person during the same or preceding week of the onset of an infection period (1) at work, (2) on business travel, (3) at home or (4) elsewhere during free time. Detailed definitions for this analysis will be created later.

#### Epidemiological research topics

The following analyses for "episodes" will all be carried out for the different infection and sick leave episodes. Detailed definitions needed for the analysis will be created later.

1) Seasonal variation of the episodes and their connection to the occurrence of infectious agents in the participating companies and, on the other hand, in Finnish population in general also utilizing molecular epidemiology of the infectious agents.

2) Distributions of the incidence and duration of episodes per person (0, 1, 2 etc episodes per person-year in relation to selected information derived from the transmission risk survey.

3) Temporal connection of the distinct episodes to each other within the clusters (optional).

## Discussion

### Potential limitations and emerged complications of the study

As mentioned in the introduction, the end-points of this trial, frequency of infectious disease episodes in office employees and that of the consequent periods of days off from work are influenced by a multitude of person-dependent and environmental factors. For some of the confounding factors attempts can be made to eliminate them or at least to minimize their effect while others cannot be regulated. Launching of the present intervention trial among the office staff of various commercial companies was coinciding with the international economical recession resulting in staff reorganization programs in some of the companies, which somewhat delayed and complicated our cluster definitions. A major challenge for the trial setup emerged after onset of the interventions, the influenza A/H1N1 pandemic that reached Finland in summer 2009 and prompted widely publicized improved hand hygiene campaigns at the national level, partly further detailed by the occupational health care units of the participating companies. Obviously, this influenced the behavior of the study participants in the control arm as well, even though it did not influence the participation percentage in the units. Some of the resulting compromises are discussed in the following.

### Lack of sample size calculations and power estimates

Although previous studies on the effects of improved hand hygiene on the occurrence of RTI and GTI appear to yield positive results in semi-closed populations, the results have been variable. We also considered that in the office work setup reintroduction of infections from outside the cluster would have a relatively greater influence than e.g. in a hospital ward. Hence, improved hygiene on the premises might have relatively less impact on the overall transmission of infections. We decided to extend the recommendation of exploiting improved hygiene at home as well, but obviously that is even more difficult to control than that in office. Therefore, we decided that sample size calculation was not possible in the lack of reliable basis for the necessary assumptions.

On the other hand, it was clear that this kind of intervention could not be randomized as an individual case-control study as cluster effects on the end points are obvious ranging from contagion at work to working team dependent traditions of symptom severity necessary to stay home. In matching the clusters we ignored, however, the company, but gave value to potential sources of contagion at home and in leisure time. We could not find any tools for determining the optimal size of a cluster. For statistical analysis in cluster randomized trials the number of people in a cluster is sometimes considered to be negatively correlated for the capacity to detect differences. On the other hand, a certain number of susceptible individuals are required for a virus to be able to circulate in a host population. We chose an arbitrary lower limit of 50 people in our study clusters. It is clear that we should have been able to recruit a larger number of clusters but because of the required operational unity and independence of distinct clusters we could not offer the recruitment to the entire staff in the dedicated companies (about 10000).

### Self reporting rather than objective signs and official records

It would be easy to criticize our study design that our data collection is based on self reporting of RTI and GTI symptoms by lay people and thus subject to potential misunderstanding, over reporting, ignorance or simplification. We cannot exclude these potential sources of inaccuracy but we have made efforts to describe the reportable symptoms in simple terms, rapidly responded to any queries and are repeating the definitions in the weekly emails requesting to send the personal report. We believe that the potential error these accuracies might cause will be evenly distributed in the three arms of the trial. On the other hand, we did not have a real alternative. As both RTI and GTI are often very mild and short lasting, most of the patients do not attend any health care services for professional assessment of the symptoms. We did not consider arranging specific study clinics to be attended by the participants at any symptoms as this kind of arrangement, on top of being operationally difficult and resource demanding, would have interfered with the routines of the regular office work too much.

Likewise, as the days off periods prompted by RTI or GTI are usually short, they are not officially recorded in all companies and in some cases it is sufficient that the absence is only notified to the immediate supervisor in a team. In short, official staff days off records are not necessarily comparable in different companies. Therefore, we decided to design a self reporting system that will give us all the desired information in a standard format.

### A need for unscheduled interim analysis, influenza A/H1N1 pandemic

Initially we planned that interim analyses will not be done. However, in the late summer 2009 the national preparation campaign to the expected 2009 H1N1-influenza pandemic caused increased use of hand washing and use of alcohol based disinfectant in the community including the control arm of this trial. When on one hand prompting volunteers in our intervention arms to maintain hygienic behaviors, the national campaign was likely to dilute the possible difference between control and intervention arms with the participants in the control clusters also increasingly adopting hand hygiene behavior. Awareness of the citizens and hand hygiene campaigns of the companies and occupational health clinics have placed control groups of this study in a different position in the autumn 2009 as compared to that in the beginning of the study from January 2009 on. In this unexpected and unavoidable situation we have decided to analyze the results in two parts.

a) The first part will cover reporting from the beginning of the study to the end of July 2009, 25 weeks.

b) The second part will include the reporting from the beginning of August 2009 to the end of the study (if nothing else remarkable appears), 43 weeks.

This will undoubtedly debase potential results as the follow-up weeks are decreased, but on the other hand, reports of the remaining follow-up weeks can be analyzed in a new set up. In the second part we will compare the effect of personal advice and demonstration-facilitated information to that of national campaign fortified by general employer instructions.

## Conclusions

Implementation of the described study means balancing between scientific conformities and feasibility in a work environment, where regular activities may not be disturbed excessively. Furthermore, it is difficult to control human behavior, even for a perceived cause. Even with these compromises, this study has potential of providing data to be utilized in future hand hygiene campaigns and infection disease control.

## Competing interests

The authors declare that they have no competing interests.

## Authors' contributions

CSK participated in the acquisition of the data, coordination of the project and analysis of the samples and data as well as drafted the manuscript. JH and PAP designed the database and participated in the study design and data analysis. TZ participated in the design of the study and coordinated the sample logistics and analysis. TK advised hand hygiene and participated in the data acquisition. PA, MH, HN, PiR and JT participated in the study design and formed connections to the participating companies. PH, MR, PeR and EV participated in the study design. TH designed the study, participated in the coordination of the project and analysis of the data as well as in the drafting of the manuscript. All authors read and approved the final manuscript.

## Supplementary Material

Additional file 1 **Contagion risk survey implemented for potential participants in the form of an electronic questionnaire**. Click here for file

Additional file 2 **Weekly report of exposure and symptoms of respiratory or gastrointestinal infection and absence from work due to infections**. The questionnaire is sent weekly to participants electronically. Click here for file

Additional file 3 **The questionnaire of behavioral habits**. Click here for file

## References

[B1] Nesti MM Goldbaum M Infectious diseases and daycare and preschool education J Pediatr (Rio J) 2007 83 4 299 312 10.2223/JPED.1649 17632670

[B2] Pittet D Allegranzi B Sax H Dharan S Pessoa-Silva CL Donaldson L Boyce JM Evidence-based model for hand transmission during patient care and the role of improved practices Lancet Infect Dis 2006 6 10 641 652 10.1016/S1473-3099(06)70600-4 17008173

[B3] Oughton MT Loo VG Dendukuri N Fenn S Libman MD Hand hygiene with soap and water is superior to alcohol rub and antiseptic wipes for removal of Clostridium difficile Infect Control Hosp Epidemiol 2009 30 10 939 944 10.1086/605322 19715426

[B4] Grayson ML Melvani S Druce J Barr IG Ballard SA Johnson PD Mastorakos T Birch C Efficacy of soap and water and alcohol-based hand-rub preparations against live H1N1 influenza virus on the hands of human volunteers Clin Infect Dis 2009 48 3 285 291 10.1086/595845 19115974

[B5] Jefferson T Foxlee R Del Mar C Dooley L Ferroni E Hewak B Prabhala A Nair S Rivetti A Interventions for the interruption or reduction of the spread of respiratory viruses (Review) The Cochrane Library 2007 4 1 54 10.1002/14651858.CD006207.pub2 17943895

[B6] Aiello AE Coulborn RM Perez V Larson EL Effect of hand hygiene on infectious disease risk in the community setting: a meta-analysis Am J Public Health 2008 98 8 1372 1381 10.2105/AJPH.2007.124610 18556606 PMC2446461

[B7] Uhari M Mottonen M An open randomized controlled trial of infection prevention in child day-care centers Pediatr Infect Dis J 1999 18 8 672 677 10.1097/00006454-199908000-00004 10462334

[B8] Ponka A Poussa T Laosmaa M The effect of enhanced hygiene practices on absences due to infectious diseases among children in day care centers in Helsinki Infection 2004 32 1 2 7 10.1007/s15010-004-3036-x 15007735

[B9] Larson EL Warned, but not well armed: preventing viral upper respiratory infections in households Public Health Nurs 2006 24 48 59 10.1111/j.1525-1446.2006.00607.x PMC7167929 17214653

[B10] Mott PJ Sisk BW Arbogast JW Ferrazzano-Yaussy C Bondi CA Sheehan JJ Alcohol-based instant hand sanitizer use in military settings: a prospective cohort study of Army basic trainees Mil Med 2007 172 11 1170 1176 18062391 10.7205/milmed.172.11.1170

[B11] Huber MA Holton RH Terezhalmy GT Cost analysis of hand hygiene using antimicrobial soap and water versus an alcohol-based hand rub J Contemp Dent Pract 2006 7 2 37 45 16685293

[B12] Graham JW Flay BR Johnson CA Hansen WB Collins LM A multiattribute utility measurement approach to the use of random assignment with small number of aggregated units Eval Rev 1984 8 247 260 10.1177/0193841X8400800206

[B13] Murray DM Varnell SP Blitstein JL Design and analysis of group-randomized trials: a review of recent methodological developments Am J Public Health 2004 94 3 423 432 10.2105/AJPH.94.3.423 14998806 PMC1448268

[B14] Ray WA Taylor JA Meador KG Thapa PB Brown AK Kajihara HK Davis C Gideon P Griffin MR A randomized trial of a consultation service to reduce falls in nursing homes JAMA 1997 278 7 557 562 10.1001/jama.278.7.557 9268276

[B15] Gelman A Carlin JB Stern HS Rubin DB Bayesian data analysis 2004 2 Chapman & Hall/CRC

