# Supplementary material for: STOPFLU: is it possible to reduce the number of days off in office work by improved hand-hygiene?
Source: Trials. 2010 Jun 4;11:69. doi: 10.1186/1745-6215-11-69 (PMC2889989; doi:10.1186/1745-6215-11-69)
Supplement: Additional file 1 — Contagion risk survey implemented for potential participants in the form of an electronic questionnaire. [file 1745-6215-11-69-S1.DOC]

Additional file 1. Contagion risk survey implemented for potential participants in the form of an electronic questionnaire.

1. Working unit

A drag down menu with all participating units

2. Sex

( )  male
( )  female

3. Age (years) ______________________________________________

POSSIBILITIES OF CONTAGION OUTSIDE WORK

4. Residents in the same household

[ ]  no school aged or younger children
[ ]  one or more school aged children
[ ]  one or more children under school age
[ ]  at least one child under school age attending day care/ preschool/ weekly club etc.
[ ]  grown-up exposed to children with cold or vomiting/diarrhea disease at work in school, kindergarten, children’s club, health care etc.

5. I use mainly public transportation for commuting to work

( )  yes
( )  no

6. I have a medically diagnosed chronic heart condition, atherosclerosis, asthma or other respiratory disease

( )  no
( )  yes, what? ______________________________________________

7. I received influenza vaccination in autumn 2008

( )  yes
( )  no
( )  I do not know

8. My work normally includes trips to other municipalities

( )  weekly
( )  monthly
( )  more rarely or not at all

9. Smoking


( )  I have never smoked
( )  I do not smoke any more
( )  I smoke in average <10 cigarettes/cigars/pipes a day
( )  I smoke in average 10-20 cigarettes/cigars/pipes a day
( )  I smoke in average >20 cigarettes/cigars/pipes a day

10. If you answered ”I do not smoke any more” to the previous question, answer to the following

| I started smoking year | ______________________ |
| --- | --- |
| I quit smoking year | ______________________ |

11. Exposure to cigarette smoke of other smokers

( )  not at all
( )  infrequently
( )  daily, how many hours a day in average ______________________________________________
