# Supplementary material for: STOPFLU: is it possible to reduce the number of days off in office work by improved hand-hygiene?
Source: Trials. 2010 Jun 4;11:69. doi: 10.1186/1745-6215-11-69 (PMC2889989; doi:10.1186/1745-6215-11-69)
Supplement: Additional file 2 — Weekly report of exposure and symptoms of respiratory or gastrointestinal infection and absence from work due to infections. The questionnaire is sent weekly to participants electronically. [file 1745-6215-11-69-S2.DOC]

Additional file 2. Weekly report of exposure and symptoms of respiratory or gastrointestinal infection and absence from work due to infections. The questionnaire is sent weekly to participants electronically.

Reported week (the past week)
Automatically given

**Working unit**

Automatically given

Exposure during the past week (I was around people who had symptoms of respiratory infection or vomiting/diarrhea disease)

|  | Exposure | | | | |
| --- | --- | --- | --- | --- | --- |
|  | at work | during work trip | at home | elsewhere during  free time | No exposure to my knowledge |
| respiratory infection | [ ] | [ ] | [ ] | [ ] | [ ] |
| vomiting/diarrhea disease | [ ] | [ ] | [ ] | [ ] | [ ] |

**Own health condition and possible absence from work during the past week**

[ ]  I was healthy and normally at work the whole week or on vacation
[ ]  I had symptoms of respiratory infection during the past week
[ ]  I had symptoms of vomiting/diarrhea disease during the past week
[ ]  I was absent from work at least one day due to another reason

Choose the most appropriate alternative for each day

|  | **"Symptoms"** mean symptoms of respiratory infection or vomiting/diarrhea disease | | | | | | | |
| --- | --- | --- | --- | --- | --- | --- | --- | --- |
|  | Healthy at work or out of work as designed | At work with symptoms | Symptoms, but not supposed to be at work | Part of the day at work with symptoms | Absent due to symptoms | Absent due to child’s symptoms; own symptoms as well | Absent due to child’s symptoms; self healthy | Other reason for absence |
| Monday | ( ) | ( ) | ( ) | ( ) | ( ) | ( ) | ( ) | ( ) |
| Tuesday | ( ) | ( ) | ( ) | ( ) | ( ) | ( ) | ( ) | ( ) |
| Wednesday | ( ) | ( ) | ( ) | ( ) | ( ) | ( ) | ( ) | ( ) |
| Thursday | ( ) | ( ) | ( ) | ( ) | ( ) | ( ) | ( ) | ( ) |
| Friday | ( ) | ( ) | ( ) | ( ) | ( ) | ( ) | ( ) | ( ) |
| Saturday | ( ) | ( ) | ( ) | ( ) | ( ) | ( ) | ( ) | ( ) |
| Sunday | ( ) | ( ) | ( ) | ( ) | ( ) | ( ) | ( ) | ( ) |
