# Supplementary material for: STOPFLU: is it possible to reduce the number of days off in office work by improved hand-hygiene?
Source: Trials. 2010 Jun 4;11:69. doi: 10.1186/1745-6215-11-69 (PMC2889989; doi:10.1186/1745-6215-11-69)
Supplement: Additional file 3 — The questionnaire of behavioral habits. [file 1745-6215-11-69-S3.DOC]

Additional file 3. The questionnaire of behavioral habits.

Work unit

A drag down menu with all participating units

**I wash my hands usually**

|  | yes | no |
| --- | --- | --- |
| having come to work | ( ) | ( ) |
| having come home from work | ( ) | ( ) |
| before eating at home | ( ) | ( ) |
| before eating at the office lunch room | ( ) | ( ) |
| before eating at a restaurant | ( ) | ( ) |

When I have a cold, usually

|  | yes | no |
| --- | --- | --- |
| I come to work, if only I can | ( ) | ( ) |
| I wash my hands more often than usually | ( ) | ( ) |
| I shake hands as usual | ( ) | ( ) |
| I cough and sneeze at my arm if I do not have time to use disposable handkerchief | ( ) | ( ) |
| I use the same disposable handkerchief only once | ( ) | ( ) |
| for smokers: I smoke as usual | ( ) | ( ) |

When I have vomiting/diarrhea disease, usually

|  | yes | no |
| --- | --- | --- |
| I come to work, if only I can | ( ) | ( ) |
| I wash my hands more often than usually | ( ) | ( ) |
| I shake hands as usual | ( ) | ( ) |

I use disinfectant hand-rub daily

|  | yes | no |
| --- | --- | --- |
| regardless of my health condition | ( ) | ( ) |
| when I have a cold | ( ) | ( ) |
| when I have a vomiting/diarrhea disease | ( ) | ( ) |

During cold seasons I usually avoid

|  | yes | no |
| --- | --- | --- |
| shaking hands | ( ) | ( ) |
| going to cinema, theater and concerts etc. | ( ) | ( ) |
| use of bus, tram and/or train depending on possibilities | ( ) | ( ) |
